# A critical role of nicotinamide phosphoribosyltransferase in human telomerase reverse transcriptase induction by resveratrol in aortic smooth muscle cells

**DOI:** 10.18632/oncotarget.3580

**Published:** 2015-03-14

**Authors:** Peixin Huang, Sean M. Riordan, Daniel P. Heruth, Dmitry N. Grigoryev, Li Qin Zhang, Shui Qing Ye

**Affiliations:** ^1^ Division of Experimental and Translational Genetics, Department of Pediatrics, Children's Mercy Hospitals and Clinics, University of Missouri Kansas City School of Medicine, Kansas City, MO, USA; ^2^ Department of Biomedical and Health Informatics, University of Missouri Kansas City School of Medicine, Kansas City, MO, USA; ^3^ Laboratory of Translational Studies and Personalized Medicine, “Northern” BioPharm Cluster at Moscow Institute of Physics and Technology, Moscow Region, Russian Federation

**Keywords:** resveratrol, NAMPT, SIRT, telomerase, aging

## Abstract

Aging is the predominant risk factor for cardiovascular diseases and contributes to a considerably more severe outcome in patients with acute myocardial infarction. Resveratrol, a polyphenol found in red wine, is a caloric restriction mimetic with potential anti-aging properties which has emerged as a beneficial nutraceutical for patients with cardiovascular disease. Although resveratrol is widely consumed as a nutritional supplement, its mechanism of action remains to be elucidated fully. Here, we report that resveratrol activates human nicotinamide phosphoribosyltransferase (NAMPT), SIRT4 and telomerase reverse transcriptase (hTERT) in human aortic smooth muscle cells. Similar observations were obtained in resveratrol treated C57BL/6J mouse heart and liver tissues. Resverotrol can also augment telomerase activity in both human pulmonary microvascular endothelial cells and A549 cells. Blocking NAMPT and SIRT4 expression prevents induction of hTERT in human aortic smooth muscle cells while overexpression of NAMPT elevates the telomerase activity induced by resveratrol in A549 cells. Together, these results identify a NAMPT-SIRT4-hTERT axis as a novel mechanism by which resveratrol may affect the anti-aging process in human aortic smooth muscle cells, mouse hearts and other cells. These findings enrich our understanding of the positive effects of resveratrol in human cardiovascular diseases.

## INTRODUCTION

Aging is the predominant risk factor for cardiovascular diseases [1] and contributes to a significantly more severe outcome in patients with acute myocardial infarction [2]. These risks are partly attributable to an age-related decline in the ability of vascular cells to resist stress and effectively remodel the arterial wall. Vascular smooth muscle cells are especially important in this regard. Strategies to prevent the premature senescence of vascular smooth muscle cells could be an effective approach for reducing vascular disease. During the past decade dietary supplementation with the plant-derived polyphenol resveratrol (3,5,4′-trihydroxystilbene) has emerged as a promising approach to counteract age-induced pro-atherogenic phenotypic changes in the vasculature. Resveratrol has been shown to exert significant anti-aging actions in vertebrates [3]. Resveratrol induced gene expression patterns resembled those induced by caloric restriction (CR) and delayed aging-related deterioration [4]. Both resveratrol and CR have beneficial effects in various mammalian models of aging and cardiovascular diseases [4, 5]. A more comprehensive review of the positive effects of resveratrol in relation to coronary artery disease can be found here [6]. Although significant progress has been achieved in elucidating the cellular mechanisms activated by resveratrol, the signal transduction pathway and mechanisms of its effect in the vascular system remains to be further elucidated.

One possible mechanism for the protective effects of resveratrol is the preservation of telomere length. Telomeres are repetitive nucleotide sequences located at the extreme ends of chromosomes and are considered to be indicators of biological age. The telomerase enzyme is responsible for the maintenance of telomeres through the addition of nucleotides. Tchirkov and Lansdorp proposed the importance of both sufficient telomerase activity and maintenance of telomere length for aging in primary human fibroblast [7]. Stimulation of the catalytic subunit of telomerase, the telomerase reverse transcriptase (TERT), stabilized telomere length and provided the cells with unlimited replicative potential without generating malignant properties [8]. Thus, telomerase reactivation can prevent or delay the cellular aging process triggered by significant telomere shortening.

Nicotinamide phosphoribosyltransferase (NAMPT), also known as Pre-B-cell colony-enhancing factor (PBEF) and Visfatin, is the rate-limiting enzyme for NAD+ biosynthesis of a mammalian salvage pathway from nicotinamide [9]. The intracellular levels of NAD+ and nicotinamide have recently been linked to cellular responses necessary for cell survival. These response pathways include members of the sirtuins, a family of protein deactylases [10]. Overexpression of NAMPT has been shown to increase SIRT1 activity [9], and protect cells from apoptosis through activation of SIRT3 and SIRT4 [11]. NAMPT has also been suggested to extend the lifespan of human vascular smooth muscle cells by activating SIRT1 and preventing the accumulation of p53 [12]. Therefore, because both resveratrol and NAMPT have been shown to be effective protective agents in the vasculature, we sought to identify a potential mechanistic link between the two agents in their anti-aging effect.

In this study, we tested the hypothesis that resveratrol may trigger hTERT, which stabilizes telomere length, in human aortic smooth muscle cells as a potential mechanism for its role in the prevention and treatment of vascular diseases. Our results indicate that resveratrol treatment indeed leads to activation of hTERT. Further investigation identified that hTERT activation is dependent upon resveratrol first inducing the expression of NAMPT followed by SIRT4. We present here evidence of a NAMPT-SIRT4-hTERT axis as the novel mechanism of anti-aging effects of resveratrol in human aortic smooth muscle cells, and propose that this pathway contributes to the positive outcomes observed in the vasculature that coincide with increased exposure to resveratrol.

## RESULTS

### Resveratrol induces hTERT and telomerase activity in human Aortic Smooth Muscle, A549, and HMVEC-L cells

**Fig. 1:**
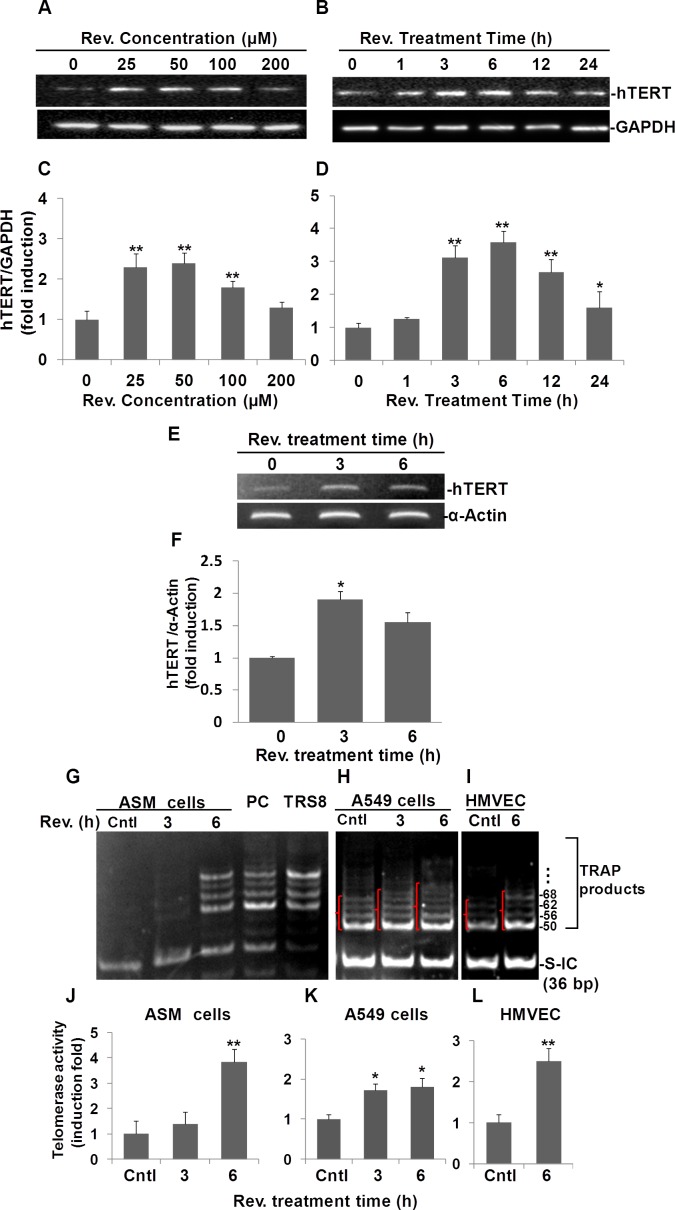
Resveratrol (Rev.) induces hTERT expression and telomerase activity in ASM cells A) Dose response of resveratrol-induced hTERT expression in ASM cells. ASM were incubated with indicated concentrations of resveratrol for 6 h. B) Time course of resveratrol-induced hTERT expression in ASM cells. ASM cells were treated with 50 μM of resveratrol for indicated times and analyzed by Western blot. C and D) Densitometry analysis of hTERT expression normalized to GAPDH and control. E) Resveratrol induces hTERT mRNA in ASM cells. ASM cells were treated with 50 μM resveratrol for the indicated times. mRNA levels of hTERT were measured by RT-PCR. α-ACTIN was probed as sample loading control. F) Densitometry analysis of resveratrol-induced mRNA level normalized to α-ACTIN. TRAP assay of telomerase activity in resveratrol treated ASM cells (G), A549 (H), and HMVEC-L cells (I). Cells were treated with 50 μM resveratrol for indicated h, then the cell lysates were tested for the telomerase activity. Lane cntls: ASM cells, or A549 cells, or HMVEC-L cells treated with equal amount of DMSO for 6h; Lanes REV. (h) 3 and 6: ASM cells (0.04 μg protein/reaction), or A549 cells (0.004 μg protein/reaction), or HMVEC-L cells (0.004 μg protein/reaction) treated with resveratrol for indicated h; Lane PC: Positive cells provided by the kit; Lane TRS8: Control template provided by the kit; J, K. L) Density of pixels in all bands above 50 bp were measured and summed. The resulting fold-change of resveratrol mediated telomerase activity normalized to internal control (S-IC) and untreaed controls. Bars represent the mean of three experiments ± SD. n≥3, *p<0.05; **p<0.01.

To examine whether resveratrol can induce hTERT expression and determine the optimum dose and time course of its potential effect in human ASM cells, we performed Western blots to assess hTERT protein levels in resveratrol treated cells compared to those in control cells. As shown in Fig. 1A and C resveratrol at concentrations of 25, 50 and 100 μM significantly induced 2.3±0.2, 2.4±0.3, 1.8±0.1 fold increases of hTERT compared to DMSO reagent treated controls, respectively. Based on this observation, we used a 50 μM dose of resveratrol for the time course experiment. As shown in Fig.1B, when we treated ASM cells with 50 μM of resveratrol over a period of 24 h, we determined that resveratrol induced hTERT expression which peaked at 6 h after treatment with a 3.6±0.3 fold increase compared to controls (Fig. 1D). Thus, we selected a dosage of 50 μM and the 6 h time point of resveratrol treatment for most of our experiments.

To examine whether the resveratrol effect is due to an increase in the levels of hTERT mRNA, we performed semi-quantitative RT-PCR analysis of hTERT mRNA in resveratrol treated human aortic smooth muscle cells. As shown in Fig. 1E and F, an increase in hTERT mRNA level was observed at 3 h post resveratrol treatment by 1.9±0.1 fold compared to untreated controls. hTERT mRNA levels were also upregulated by 1.6±0.2 fold compared to control after 6 h of resveratrol treatment. These observations were validated further by qRT-PCR analyses showing that resveratrol upregulated hTERT expression to 2.3±0.1 and 3.6±0.5 fold after 3 and 6 h, respectively. (Fig. S1B). These results suggest that upregulation of hTERT by resveratrol is mainly via its effect on the increase of hTERT mRNA level.

Moreover, we applied the TRAPeze Gel-Based Telomerase Detection Assay to check if resveratrol increases telomerase activity. The resulting gel image from ASM cells (Fig. 1G), shows that the 36-bp standard internal control band (S-IC) is observed with similar pixel density in every lane except the TRS8 template control lane, suggesting a high efficiency of PCR amplification and an equal amount of protein analyzed for each reaction. The cell lysate for the telomerase positive control cells (provided with the kit), lane PC, showed a ladder pattern of PCR products with 6-base increments starting at 50 nucleotides (i.e. 50, 56, 62, 68, and so forth). Cells were considered positive for telomerase activity when a 36-bp internal control band and a ladder of PCR products with 6-base increments similar to that of the telomerase-positive control lane were present (Fig. 1G, Lane PC). There were no obvious TRAP product ladders detected in untreated ASM cells (Lane Cntl). However, after ASM cells were treated with 50 μM resveratrol for 3 h, the lane showed a very weak ladder pattern. After 6 h treatment, the TRAP products were observed clearly, similar to the positive cells. Our TRAPeze assay data indicated that resveratrol increases telomerase activity in ASM cells, especially at the 6 h treatment time point.

Increased telomerase activity was also observed in A549 and HMVEC-L cells (Fig. 1H and I). Unlike ASM cells, in which telomerase activity in controls was undetectable, untreated A549 cells and HMVEC-L cells were positive for telomerase activity. However, after treatment with resveratrol for 3 and 6 h, the telomerase activities in A549 cells increased in a time-dependent manor (Fig. 1H). In addition, increased telomerase activity was also observed in HMVEC-L cells after 6 h of treatment with resveratrol (Fig. 1I).

Pixel densities within all DNA bands above 50 bp for each sample were measured to quantify the resveratrol induced telomerase activity. Our data showed that telomerase activity was increased significantly to 3.8±0.3, 1.7±0.2 and 2.5±0.3 fold following 6 hrs of reveratrol treatment relative to DMSO treated controls in ASM (Fig. 1J), A549 (Fig. 1K), and HMVEC-L (Fig. 1L) cells, respectively.

The above data collectively suggest that resveratrol not only induced human TERT both at the protein and mRNA levels in ASM cells, but also increased telomerase activity in ASM, A549, and HMVEC-L cells.

### Resveratrol induces NAMPT in human Aortic Smooth Muscle Cells

**Fig. 2:**
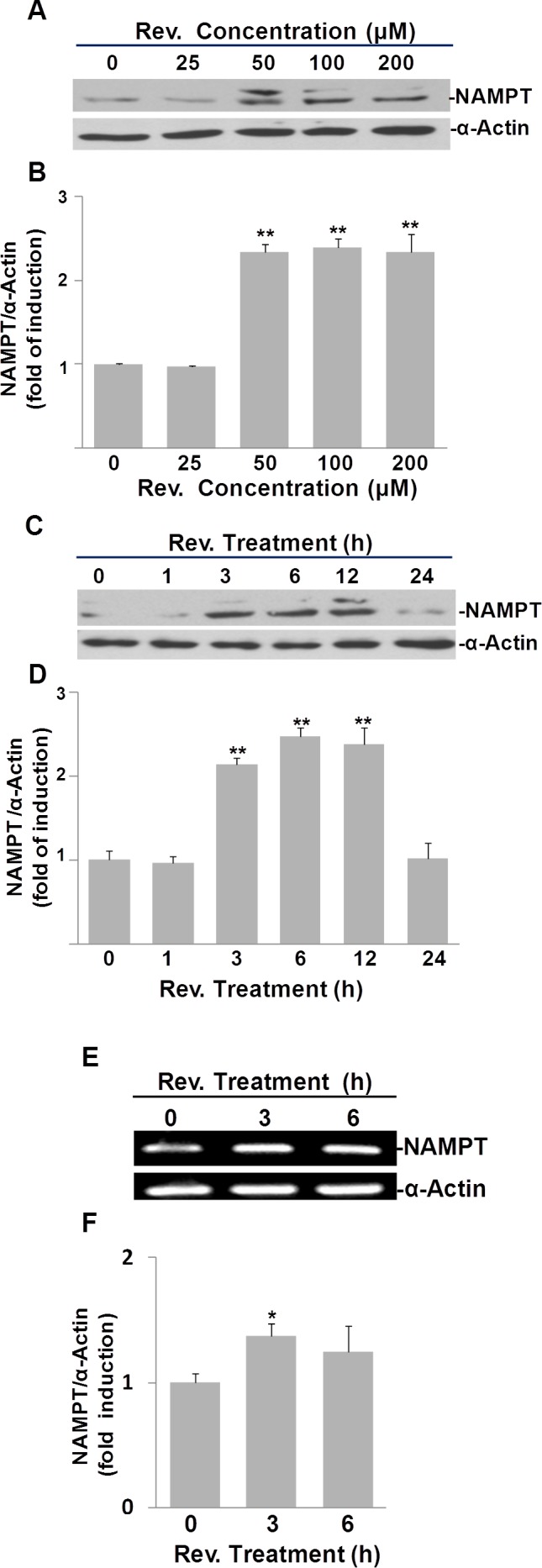
Resveratrol (Rev.) induces NAMPT expression in ASM cells A) Dose response of resveratrol-induced NAMPT expression in ASM cells. Cells were incubated with indicated concentrations of resveratrol for 6 h and analyzed by Western blot. B) Densitometry analysis of resveratrol-induced NAMPT expression normalized to α-Actin and untreated controls. C) Time course of resveratrol-induced NAMPT expression in ASM cells. Cells were treated with 50 μM of resveratrol for indicated times and analyzed by Western blot. D) Densitometry analysis of resveratrol-induced NAMPT expression normalized to α-Actin and untreated controls. E) Resveratrol induces NAMPT mRNA in ASM cells. ASM cells were treated with 50 μM resveratrol for the indicated times. mRNA levels were measured by RT-PCR. F) Densitometry analysis of resveratrol-induced NAMPT mRNA normalized to α-Actin and untreated controls. Bars represent the mean of three experiments ± SD. *p<0.05; **p<0.01.

To investigate whether resveratrol also induces NAMPT expression, we quantified NAMPT protein in resveratrol treated ASM cells. First, we performed a dose-response experiment (Fig. 2A). Treating cells with 50, 100, 200 μM of resveratrol significantly elevated cellular NAMPT protein levels to 2.3±0.1, 2.4±0.1, and 2.3±0.2 fold changes, respectively, compared to DMSO controls (Fig. 2A and 2B). Second, we performed a time course experiment over 24 h using 50 μM of resveratrol (Fig. 2C). Our results show an increase in NAMPT beginning at 3 h and reverting to the baseline by 24 h post treatment. Resveratrol treatment induced positive fold changes in NAMPT of 2.1±0.1, 2.5±0.1, and 2.4±0.2 compared with untreated controls at 3, 6, and 12 h time points, as evaluated by pixel density, respectively (Fig. 2D). Thus, we also selected a dosage of 50 μM and a 6 h time point of resveratrol treatment for the subsequent experiments.

To examine whether resveratrol's effect on NAMPT protein expression occurs at the mRNA level, we performed semi-quantitative RT-PCR analysis of NAMPT mRNA in resveratrol treated human ASM cells. In line with our Western blotting results, our RT-PCR results clearly showed that at both 3 h and 6 h time points, NAMPT mRNA was induced by resveratrol, particularly at the 3 h time point (Fig. 2E), where there was a 1.5±0.1 fold increase compared to DMSO controls (Fig. 2F). These observations were validated further by qRT-PCR analyses, as resveratrol upregulated NAMPT expression 1.5±0.3 and 1.7±0.5 fold after 3 and 6 hours, respectively, compared to untreated ASM cells (Fig. S1A).

These results suggest that upregulation of NAMPT by resveratrol is mainly via its effect on the increase of NAMPT mRNA levels rather than post-translational regulation of protein level.

### Resveratrol-induced hTERT expression in ASM cells requires NAMPT

**Fig. 3:**
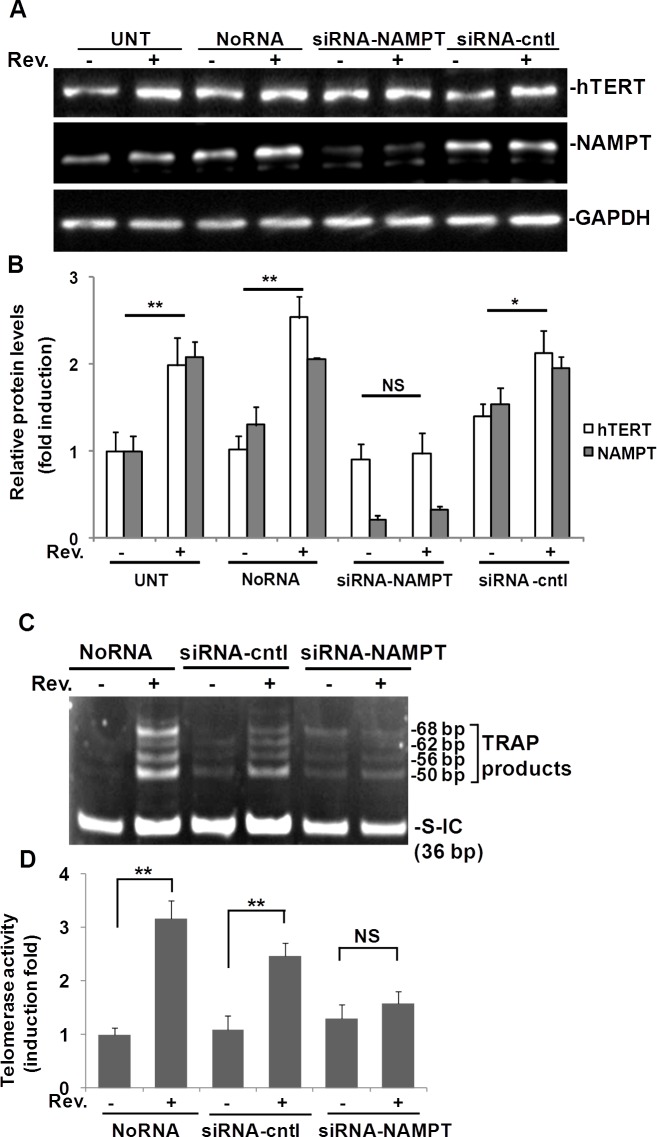
Resveratrol (Rev.)-induced hTERT and telomerase activity in ASM cells requires NAMPT ASM cells were transfected with 100 nM of NAMPT siRNA (siRNA-NAMPT) and then treated with 50 μM resveratrol for 6 h. A) Western blot for hTERT, NAMPT and GAPDH (loading control), respectively. B) Densitometry analysis of resveratrol-induced hTERT normalized to GAPDH and untreated controls. C) Representative image of telomerase activity assay (0.04 μg protein/reaction). D) For each sample, the density of pixels in all bands above 50 bp were measured and summed and analyzed with the signal normalized to internal control (S-IC) and untreated controls. Bars represent the mean of three experiments ± SD. **p<0.01; *p<0.05; NS: no significant difference.

To address whether resveratrol-induced hTERT is NAMPT-dependent, we transfected ASM cells with NAMPT siRNA to knock down NAMPT and then assessed whether resveratrol could still induce hTERT expression in these cells. In this experiment, we used 4 groups of ASM cells as shown in Fig. 3: Untreated control group (UNT), transfection reagents control group (NoRNA), NAMPT knocked down with siRNA group (siRNA-NAMPT), and siRNA control group (siRNA-cntl). NAMPT was knocked down approximately 90% using NAMPT siRNA (Fig. 3A). In the NAMPT-siRNA group, resveratrol's ability to elicit an increase in hTERT expression was abolished (Fig. 3A). Resveratrol induced a 2.0±0.2 fold increase of hTERT, and a 2.1±0.2 fold increase of NAMPT in the untreated group of cells compared to the DMSO control. Similar results were observed in the No RNA group of cells (Fig. 3B). Resveratrol induced a 1.5±0.3 fold increase of hTERT, and a 1.5±0.1 fold increase of NAMPT in the siRNA-cntl group, but hTERT was not significantly increased in siRNA-NAMPT treated cells (Fig. 3B).

Telomerase activity was also measured in ASM cells in which NAMPT was knocked down (Fig. 3C). In line with our data (Fig.1G), in no-siRNA treated cells, telomerase activity was not detected, but after treatment with resveratrol for 6 h, the TRAP bands appeared. In siRNA-cntl cells, resveratrol treatment increased telomerase activity. However, in the cells transfected with siRNA against NAMPT, resveratrol did not elevate telomerase activity to the same level as in siRNA-control cells (Fig. 3C). Quantification of pixel densities within bands above 50 bp showed that telomerase activity was elevated 2.4±0.4; 2.1±0.2, and 1.1±0.2 fold following 6 hr resveratrol treatment relative to the corresponding DMSO treated controls in noRNA, siRNA control, and siRNA-NAMPT transfected ASM cells, respectively (Fig. 3D).

Our data suggest that NAMPT is necessary for resveratrol-induced hTERT expression and telomerase activity in ASM cells.

### NAMPT overexpression elevated resveratrol-increased telomerase activity in A549 cells

**Fig. 4:**
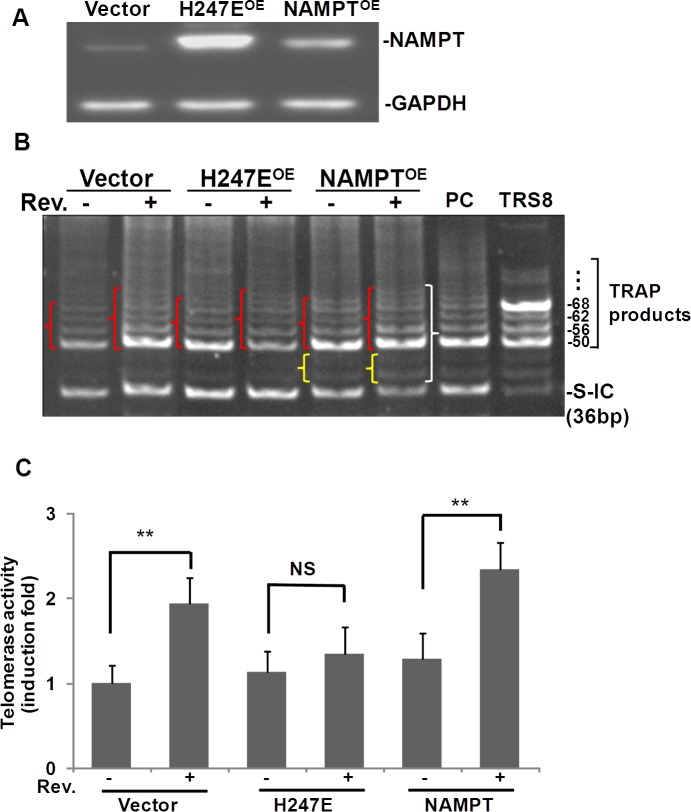
Overexpression of NAMPT, not NAMPPT-H247E elevated Rev-induced hTERT in A549 cells A549 cells were transfected with plasmids of pCAGGS, pCAGGS-NAMPT or pCAGGS-NAMPT-H247E for 42 h, and then cells were treated with or without resveratrol for 6 h. A) Western blotting of overexpression of NAMPT by transfection of pCAGGS-(vector), pCAGGS-H247E (H247E^OE^), and pCAGGS-NAMPT (NAMPT^OE^). B) Representative image of TRAP assay of telomerase activity. PC: positive control cells provided by the kit. TRS8: TRAP template control. C) For each sample, the density of pixels in all bands labeled in } were summed and analyzed with normalized to internal control (S-IC) and untreated controls. Bars represent the mean of three experiments ± SD. **p<0.01; NS: no significant difference.

To explore further the role of NAMPT in resveratrol-induced hTERT, A549 cells were transfected with pCAGGS-vector (vector control), pCAGGS-NAMPT or pCAGGS-NAMPT-H247E, a mutant NAMPT without enzymatic activity (13). NAMPT levels were elevated in A549 cells transfected with either pCAGGS-NAMPT or pCAGGS-NAMPT-H247E compared to the pCAGGS transfection control (Fig. 4A). Cells transfected with the H247E construct presented with NAMPT levels 2 times higher than in cells transfected with pCAGGS-NAMPT (Fig. 4A). We then performed the telomerase activity assay on protein extracts isolated from the transfected cells (Fig. 4B). Our data showed that in H247E transfected cells (H247E^OE^), resveratrol elevated telomerase activity slightly, compared to the resveratrol-untreated cells. In the case of the pCAGGS-NAMPT transfected cells (NAMPT^OE^), larger, more intense TRAP product bands were observed, along with additional bands between 36 bp and 50 bp in the cells treated with resveratrol compared to both the untreated pCAGGS-NAMPT cells and the resveratrol treated, pCAGGS-vector control cells. According to the telomerase activity detection kit's manual, the extra bands appearing between 36 and 50 bp suggests high telomerase activity.

Pixel densities within bands (shown in { in Fig. 4B) were measured and the telomerase activity was induced by resveratrol to 1.8±0.3 fold of controls in A549 cells transfected with empty vector, while in the cells transfected with either H247E or NAMPT expression vectors, resveratrol induced 1.3±0.3 and 2.4±0.4 fold increase in telomerase activity compared to DMSO treated controls, respectively (Fig. 4C). These findings suggested telomerase activity was increased further when NAMPT was overexpressed.

These data suggest a pivotal role for NAMPT in resveratrol-induced hTERT expression and telomerase activity in both ASM and A549 cells.

### Resveratrol elevated the NAD+ plus NADH levels in ASM cells

Intracellular NAMPT is an essential and rate-limiting enzyme in a key mammalian NAD+ biosynthetic salvage pathway. As our data showed, resveratrol induced NAMPT in ASM cells. Therefore, we tested the effects of resveratrol on NAD^+^ plus NADH levels in ASM cells (Fig. S2). ASM cells treated with 50 μM resveratrol for 3, 6, and 24 h increased intracellular NAD^+^ plus NADH levels significantly, with a peak at the 6 h treatment time point. At the 1 h treatment time point, resveratrol did not show obvious effects on NAD^+^ plus NADH levels in ASM cells (Fig. S2).

### Resveratrol regulates members of the SIRT family in ASM cells

**Fig. 5:**
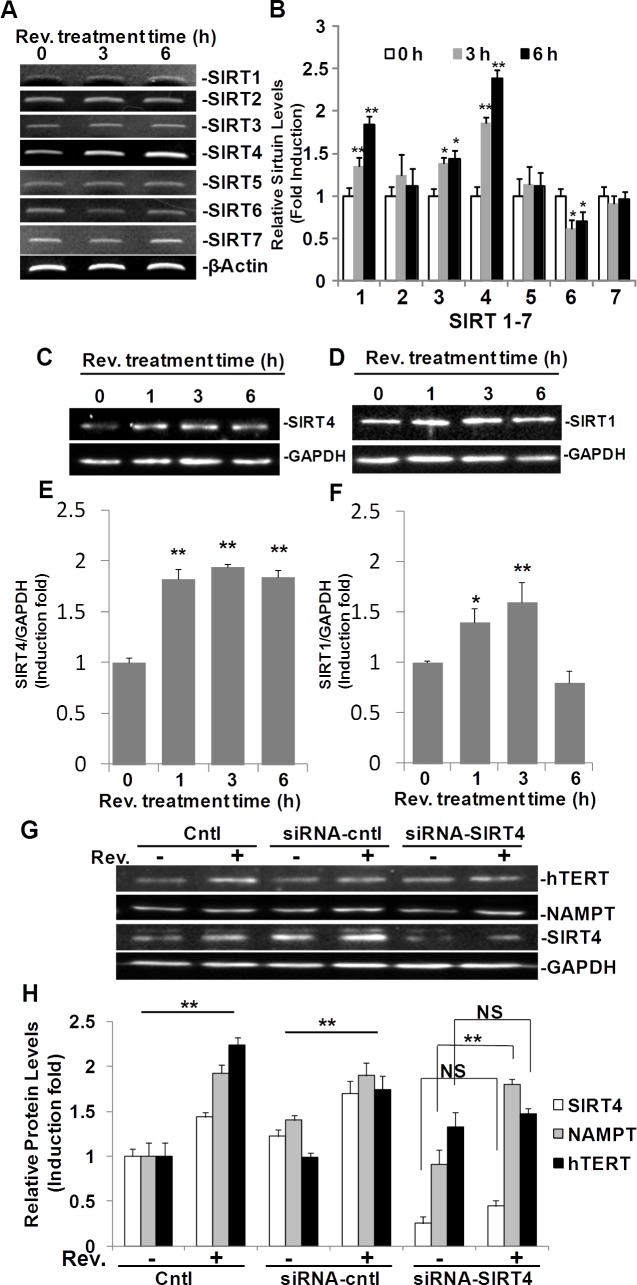
SIRT4 is regulated by resveratrol and is required in resveratrol-induced hTERT in ASM cells ASM cells were treated with 50 μM resveratrol for 3 or 6 h. A) mRNA levels were measured by RT-PCR. B) Densitometry analysis of SIRT1-7 mRNA normalized to GAPDH and untreated controls. C) Western blot for SIRT4 expression. D) Western blot for SIRT1 expression. E and F) Densitometry analysis of SIRT4 and SIRT1 expression normalized to GAPDH and untreated controls. G) Representative western blotting images of hTERT, NAMPT, SIRT4, and GAPDH protein levels in ASM cells with SIRT4 knocking down by siRNA-SIRT4. H) Densitometry analysis of protein expression normalized to GAPDH and untreated controls. Bars represent the mean of three experiments ± SD. **p<0.01; *p<0.05; NS: no significant difference.

Since NAMPT is known to regulate the SIRT family of NAD dependent deacetylase genes [11, 13], we investigated further whether resveratrol regulates SIRT1-7 in ASM cells as a possible link between NAMPT and hTERT. Specific primers for SIRT1 through SIRT7, spanning at least one intron, were designed as shown in Table 1, and semi-quantitative RT-PCR was performed in resveratrol treated ASM cells. Our data show that after 3 h of exposure to resveratrol (50μM), mRNA levels of SIRT1, SIRT3, and SIRT4, were upregulated while SIRT6 was down-regulated (Fig. 5A). Expression of SIRT 2, 5 and 7 seemed unaffected by this dose of resveratrol. Among these upregulated SIRT genes, SIRT4 was the most highly expressed following resveratrol treatment. SIRT4 mRNA levels were elevated 1.9±0.1 and 2.4±0.1 fold compared to the DMSO treated controls at 3 h, and 6 h, respectively (Fig. 5B). These observations were validated further by TaqMan qRT-PCR analyses (Fig. S1D). SIRT3 expression was increased by 1.4±0.1 and 1.5±0.1 fold compared to the DMSO control after 3 and 6 hours, respectively (Fig. 5B). The SIRT1 gene was upregulated by 1.3±0.1 and 1.6±0.1 after 3 and 6 hours, respectively (Fig. 5B). The expression of SIRT3 and SIRT1 mRNA were validated further by TaqMan qRT-PCR analyses showing that resveratrol upregulated SIRT1 expression 1.6±0.1 and 1.6±0.2 fold after 3 and 6 hours, respectively, compared to untreated ASM cells (Fig. S1C). Protein levels of SIRT1 and SIRT4 were also investigated in response to resveratrol, as SIRT4 was the most modulated protein and SIRT1 was reported to be target of resveratrol (16). Western blot results showed that resveratrol induced SIRT4 after 1, 3, and 6 h treatment time and peaked at 3 h post treatment with a 1.9±0.1 fold change increase over untreated controls (Fig. 5C and 5E), while SIRT1 was induced by resveratrol at a lower level than SIRT4 (Fig. 5D and 5F).

**Table 1:** primers and products

Name	Accession No.	5′ Primers	3′ Primers	Product size (bp)
hTERT	NM_198253.2	GTGACCGTGGTTTCTGTGTG	TCGCCTGAGGAGTAGAGGAA	214
NAMPT	NM_005746	AAGCTTTTTAGGGCCCTTTG	AGGCCATGTTTTATTTGCTGACAAA	319
SIRT1	NM_012238.4	ACGCTGGAACAGGTTGCGGGA	AAGCGGTTCATCAGCTGGGCAC	167
SIRT2	NM_012237	ATCCACCGGCCTCTATGACAA	CGCATGAAGTAGTGACAGATGG	161
SIRT3	NM_012239.5	CGGCTCTACACGCAGAACATC	CAGCGGCTCCCCAAAGAACAC	226
SIRT4	NM_012240.2	GCCTCA ATTCTCCTCCCACC	ACAGGACCCTGTCCATGC	166
SIRT5	NM_031244.3	GCCATAGCCGAGTGTGAGAC	CAACTCCACAAGAGGTACATCG	156
SIRT6	NM_016539.2	CCCACGGAGTCTGGACCAT	CTCTGCCAGTTTGTCCCTG	193
SIRT7	NM_016538.2	GCCTGAAGGTTCTAAAGAAGTACC	GTCGCCAGTGAGAAAATGGG	225
ACTIN	NM_001101	CAAACATGATCTGGGTCATCTTCTC	GCTCGTCGTCGACAACGGCTC	487

To test the hypothesis that upregulation of hTERT is also SIRT4 dependant, we knocked down SIRT4 in ASM cells using SIRT4 siRNA. SIRT4 was knocked down approximately 80% by its cognate siRNA. Accordingly, the effect of resveratrol on hTERT induction was attenuated (Fig. 5G and 5H). Furthermore, we explored the relationship between NAMPT and SIRT4 in this pathway. SIRT4 knock down attenuated resveratrol mediated hTERT upregulation but did not detectably affect resveratrol mediated NAMPT upregulation (Fig. 5H). These results suggested that in addition to NAMPT, resveratrol-induced hTERT is also SIRT4 dependent and SIRT4 is down-stream of NAMPT in this process.

### Resveratrol-induced Tert, Nampt, Sirt4, and telomerase activity in mice

**Fig. 6:**
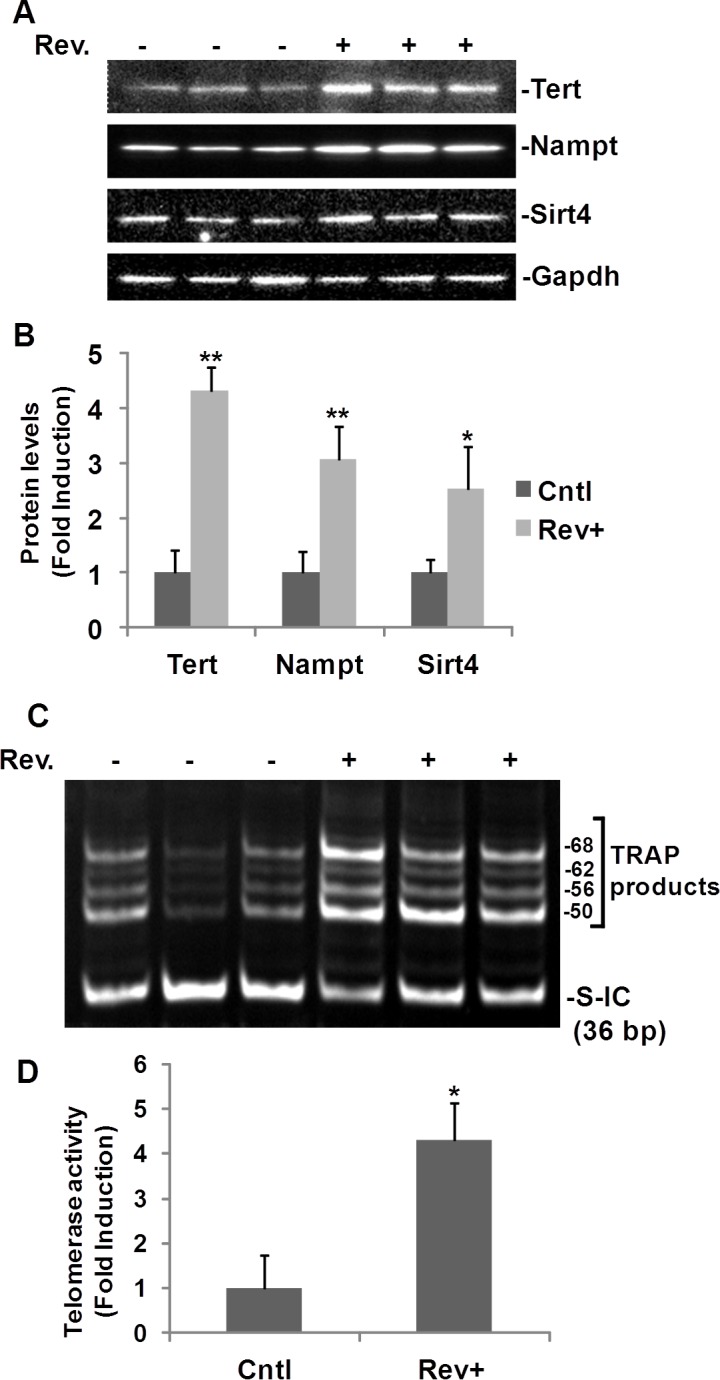
Resveratrol-induced TERT and telomerase activity in mouse heart C57 BL/6J mice (11-12 week old, male) were intraperitoneally injected with resveratrol (30 mg/kg body weight) or with DMSO (vehicle) once a day for ten days. Hearts (25-50 mg) were homogenized in lysis buffer. A) Western blot analyses of Tert, Nampt, and Sirt4. B) Densitometry analysis of resveratrol induced protein expression normalized to Gapdh and untreated controls. C) Representative image of TRAP telomerase activity assay for 0.04 μg of mouse heart protein. D) Densitometry analysis of resveratrol mediated telomerase activity normalized to the internal control (S-IC) and untreated controls. For each sample, the density of pixels in all bands above 50 bp were summed and analysed. Bars represent the mean mean ± SD. *p<0.05, **p<0.01.

To validate our findings further, we assayed protein expression and telomerase activity in mice treated with resveratrol. Male mice (11-12 weeks old, n=6) were randomly assigned to either the resveratrol treated group (n=3) or the DMSO treated control group (n=3). Mouse heart lysates were used for both Western blot analyses and telomerase activity assays (Fig. 6). In line with our *in vitro* results, Western blotting showed that expression of Tert, Nampt and Sirt4 were greatly induced in the hearts of mice treated with resveratrol (Fig. 6A). Compared with the control group, Tert, Nampt, and Sirt4 levels increased 4.3±0.4, 3.1±0.6, and 2.5±0.8 fold, respectively, in resveratrol treated mice compared to DMSO treated mice (Fig. 6B). Telomerase activity was also increased in heart tissue from resveratrol treated mice compared to DMSO vehicle treated control mice (Fig. 6C). In 3 control mice, we observed 4 weak TRAP product bands in each sample, while in resveratrol treated mice, all of the TRAP product bands were brighter and more dense, which suggested higher telomerase activity in the hearts of resveratrol treated mice (Fig. 6C). The mean levels of telomerase activity in resveratrol treated mice were 4.3±0.8 fold higher than the activity in hearts isolated from DMSO treated control mice, as evaluated by measuring pixel density within bands above 50 bp (Fig. 6D).

Telomerase activity assays and Western blotting were also performed on liver tissue isolated from C57BL/6J female mice of different ages. Telomerase activity decreased with age in resveratrol untreated mice (Fig. S3A). Using 0.04 μg protein for the assay, we clearly observed more TRAP bands in 6-week aged mouse liver than 11-week aged mouse liver, and more TRAP bands were observed in 11-week aged mouse liver than 27 week-aged mouse liver (Fig. S3A). In the 27-week aged mouse, we detected stronger TRAP bands in resveratrol treated mouse than untreated two mice (Fig. S3B). Because in 11-week aged mice, the telomerase activities were too high that most of bands appeared between 36 bp internal control and 50 bp bands in resveratrol treated mice (data not shown). In order to clearly show the effects of resveratrol, we then diluted the lysate 10 times in this group of miceand performed the telomerase activity assays again. Our data showed that resveratrol induced a noticeable increase in telomerase activity in 11-week aged mice (Fig. S3C). Both the number and the pixel density of TRAP bands above 50 bp were increased in resveratrol treated mice than untreated mouse (Fig. S3C). Western blot analysis of liver lysates further strengthened our findings (Fig.S3D). Tert expression decreased with age in untreated mouse liver. Resveratrol induced Tert expression in both mice at age of 11-weeks. In 27-week aged mice, resveratrol failed to induce an increase in Tert expression as significantly as in the 11-week aged mice, which is in line with our results from the telomerase activity assay (Fig. S3B and C). Furthermore, resveratrol induced both Nampt and Sirt4 in the 27-week and 11-week aged mice (Fig. S3D).

Our data strongly suggests that resveratrol induced telomerase activity, along with Tert, Nampt, and Sirt4 expression *in vivo*.

## DISCUSSION

The positive health effects of resveratrol on age-related diseases, such as cancer, diabetes, and cardiovascular diseases, have become increasingly appreciated in recent years [14]. There are numerous known targets of resveratrol linked to its cardioprotective activity, as previously reviewed [15], however, the details about many of these signal transduction mechanisms involved in these processes remain to be elucidated fully. In this study we demonstrate that resveratrol activates the human TERT gene, which is generally considered to be a longevity gene in mammalian cells. We established further that this activation is dependent upon enhanced NAMPT and SIRT4 expression. These findings implicate a NAMPT-SIRT4-hTERT axis as a fundamental determinant of resveratrol action in human aortic smooth muscle cells.

Both cardiovascular risk factors and common cardiovascular diseases, such as atherosclerosis, heart failure, and hypertension, are associated with short leukocyte telomeres [16]. In our study system, resveratrol greatly increased activation of hTERT in ASM cells. Resveratrol also induced higher telomerase activity in ASM and HMVEC-L cells, relative to the induction in A549 cells, which already had high levels of telomerase activity (Fig. 1G, 1H, and 1I). In conjunction with previous studies that resveratrol activates hTERT in endothelial progenitor cells [17], but down-regulates hTERT in breast cancer cells [18], our findings may suggest resveratrol acts differently in normal cells compared to cancer cells. Further studies are needed to determine the validity of this idea.

We utilized the sensitive TRAP-eze assay system to detect telomerase activity in lysates isolated from resveratrol treated cells. We showed that 50μM of resveratrol increased TRAP products significantly, especially at the 6 h treatment time point not only in ASM cells, but also in A549 cells and HMVEC cells (Fig. 1G, 1H, and 1I), strongly suggesting resveratrol increases telomerase activity in ASM, A549, and HMVEC cells. These data support the idea that resveratrol induction of hTERT results in increased telomerase activity.

The role of NAMPT as a driver of cellular stress resistance and longevity is noteworthy in the context of its expression profile. NAMPT is upregulated by stressful stimuli including infection and pro-inflammatory cytokines [19-21]. Recent studies also identify NAMPT as underlying an aging suppression pathway in smooth muscle cells, with potential relevance to controlling atherosclerosis and possibly other diseases of aging [22]. In this study, we first report that resveratrol increases both NAMPT mRNA and protein (Fig. 2) in ASM cells. Furthermore, we found that treatment with 50 μM resveratrol for 6 hours also increased intracellular NAD^+^ plus NADH levels significantly in ASM cells, presumably via the upregulation of NAMPT, which is a key enzyme in NAD synthesis (Fig. S2).

The NAD-dependent protein deacetylase sirtuin (SIRT) family of proteins promotes cell survival, stress resistance, and longevity as reviewed by Guarente [23]. The majority of our knowledge of the SIRT family pertains to SIRT1, though the functions for the other SIRT genes, SIRT2–7, in mammalian physiology have recently emerged. Decreased expression of SIRT1 is associated with endothelial dysfunction in arteries from aged mice and humans [24]. It is generally accepted that resveratrol is a SIRT1 activator [12]. In this study, we explored SIRT1-7 using semi-quantitative RT-PCR, and our results showed resveratrol up-regulates SIRT1, SIRT3, SIRT4, and down-regulates SIRT6 in ASM cells (Fig. 5A and B). Of these four genes, SIRT4 was the most highly upregulated. In accordance with these results, protein levels of SIRT4 and SIRT1 in resveratrol treated ASM cells were also enhanced (Fig. 5C and D). The induction of both SIRT4 and SIRT1 were validated further by qRT-PCR (Fig. S1D and C). We then investigated if NAMPT, and SIRT4 were required for resveratrol-induced hTERT. Transfection of ASM cells with siRNA specific for NAMPT and SIRT4 reduced expression by 90% and 80%, respectively. In both instances when NAMPT or SIRT4 were knocked down, resveratrol induction of hTERT was blocked (Fig. 3A and B; Fig. 5G and H), suggesting NAMPT and SIRT4 are necessary for resveratrol induction of hTERT. Meanwhile knockdown of SIRT4 did not block resveratrol induction of NAMPT (Fig. 5G), suggesting that NAMPT functions upstream of SIRT4. We also explored the role of SIRT1 in this process, but we did not observe the significant difference in hTERT induction between ASM cell and SIRT1 knocking down ASM cells (data not shown). Our results is in line with the other group showing that NAMPT-mediated cell protection from stress–induced cell death requires SIRT3 and SIRT4, not SIRT1 [11].

To strengthen our findings that NAMPT plays a pivotal role in resveratrol-induced hTERT and telomerase activity, we transfected A549 cells with pCAGGS-NAMPT (NAMPT^OE^) and pCAGGS-NAMPT-H247E (H247E^OE^). pCAGGS-H247E is a mutated form of NAMPT and its overexpression does not have enzymatic activity. In our system, although A549 cells transfected with H247E expressed more NAMPT protein than the pCAGGS-NAMPT transfected cells (Fig. 4A). The telomerase activity was not increased by resveratrol as high as in the cells transfected with NAMPT (Fig. 4B and C), suggesting that NAMPT enzymatic activity is crucial in resveratrol-induced hTERT expression and telomerase activity.

We observed that the baseline of hTERT expression and telomerase activity were increased slightly by siRNA-NAMPT (Fig. 3A and B), which may be due to the side effects of the transfection reagents. To understand fully the effect of resveratrol-induced telomerase activity, we performed animal experiments in our study. As a preliminary experiment, we treated a few mice at different age groups. After treating the mice with resveratrol (30 mg/kg) or the same volume of DMSO (vehicle control) for ten days, we performed telomerase activity assays and Western blot analyses on mice livers. The telomerase activities in untreated mice livers were shown to decrease with age, suggesting the high reliability and efficiency of our experimental system (Fig. S3A). Although we did not examine a large sample size in every age group, our findings suggested that resveratrol induced telomerase activity and Tert expression are related with age. Increased telomerase activity induced by resveratrol was detected more obviously in 11-week old mice compared to the 27-week old mice both for telomerase activity assays and for Western blot analysis (Fig. S3). Thus, we then used 11-12 weeks of aged male C57 BL/6J mice and performed telomerase activity assays and Western blot analyses on heart tissues, as the benefit of resveratrol on cardiovascular system is the major goal of our study. Our *in vivo* data strongly strengthened our *in vitro* findings (Fig. 6). We observed weaker bands in untreated three mice, and after treatment with resveratrol, the telomerase activities were elevated in all 3 mice hearts as the TRAP product bands are all much denser and brighter (Fig. 6C and D). Western blot analyses also showed that resveratrol induced Tert, Nampt, and Sirt4 protein expression in the hearts of resveratrol treated mice (Fig. 6A and B).

Moving forward we hope to elucidate further the connection between additional SIRT family members and the described resveratrol pathway. Natural targets of investigation include overexpression of NAMPT and SIRT4 *in vitro.* It would also be interesting to investigate whether SIRT1 plays a role in this pathway even though it was not as highly affected by resveratrol as SIRT4.

Cell senescence, as well as the decline of tissue regenerative potential, are strongly implicated in age-related pathologies as previously reviewed [25]. In addition, vascular smooth muscle cell senescence, a key feature of atherosclerotic lesions [26], can be especially dangerous because the resulting proinflammatory and non-reparative condition can induce both lesion disruption and acute vascular occlusion. Our findings show that resveratrol could activate human telomerase via a NAMPT and SIRT4 dependent pathway. Thus, we propose a NAMPT-SIRT4-hTERT axis, as illustrated in Fig. S4, which may represent a novel signal transduction mechanism for the anti-aging effects of resveratrol in human ASM cells enriching our understanding for how resveratrol contributes to positive outcomes in human cardiovascular diseases. Our validated observations in resveratrol treated mouse heart tissues, HMVEC-L cells, and A549 cells suggest that “NAMPT-SIRT4-hTERT axis” may be a generalized mechanism underlying the reseveratrol's beneficial role in human physiology.

## MATERIALS AND METHODS

### Cells

Primary human Aortic Smooth Muscle cells (ASM cells) were purchased from ATCC (Catalog #: PCS-100-012, Manassas, VA), and maintained in vascular cell basal medium (Catalog #: PCS-100-030, ATCC, Manassas, VA), completed with vascular smooth muscle cell growth kit (Catalog #: PCS-100-042, ATCC, Manassas, VA). ASM cells were passaged twice a week and used between passages 5 and 8. Human A549 cells were obtained from ATCC (cat. no. CCL-185^TM^, Manassas, VA) and maintained in Dulbecco's modified Eagle's medium with 10% fetal bovine serum. Human Microvascular Endothelial Cells - Lung (HMVEC-L) (Catalog #: CC-2527, Lonza, Rockland, ME) were cultured in endothelial cell (EC) growth medium (EBM-2; Catalog #: CC-3156, Lonza), supplement with EGM-2 SingleQuots (Catalog #: CC-4176, Lonza). HMVEC-L were studied at passages 5–8. For experiments, the cells (3×10^5^) were seeded into 6-well plates, incubated at 37°C overnight to reach 95% confluence, and then treated with the indicated concentrations of resveratrol or siRNA reagents.

### Antibodies and chemicals

Resveratrol (Catalog #: R5010) was from Sigma-Aldrich, St Louis, MO) was dissolved in DMSO (Catalog#: D2438, Sigma-Aldrich, St Louis, MO) for a stock solution of 200 mM. Resveratrol, or an equal volume of DMSO as a vehicle control, was added into 6-well plates (2 ml medium per well), for a final concentration of 200, 100, 50, or 25 μM.

The following antibodies and their dilutions were used for this study: rabbit polyclonal anti-human PBEF (NAMPT) from Bethyl Laboratories, Inc. (Catalog#: A300–372A, Montgomery, TX) (1:4,000); rabbit polyclonal anti-human TERT (hTERT) from Calbiochem (Catalog#: 582000, San Diego, CA) (1:1,000); rabbit polyclonal anti-human SIRT4 from Sigma-Aldrich (Catalog#: SAB2103969, St Louis, MO) (1:1000); rabbit polyclonal anti-human SIRT1 (H-300) (Catalog#: Sc-15404; 1:1000) and donkey anti-rabbit IgG-HRP from Santa Cruz Biotechnology (Catalog#: sc-2313, Santa Cruz, CA) (1:4000).

### RNA isolation

Semi-quantitive RT-PCR, qRT-PCR. Details of total RNA isolation and Semi-quantitive RT-PCR, qRT–PCR are provided in Supporting Information (S1).

### Western blotting

Western blot analysis was performed as described previously [27].

Telomerase Activity Assay. Telomerase activity was determined using the TRAPeze telomerase detection kit (Catalog#: S7700, Millipore, Billerica, MA). The details were given in Supporting Information (S1).

NAD^+^ plus NADH Assay. The NAD^+^ plus NADH levels in the cell lysates were tested using a commercial kit (Catalog#: 600480, Cayman Chem. Co., Ann Arbor, MI) and performed according to the protocol provided by the manufacturer.

### siRNA, Recombinant Human NAMPT and Mutant NAMPT Transfection

Details of siRNA, plasmids of Recombinant Human NAMPT Mutant NAMPT, and their transfection are provided in Supporting Information (S1).

### Animal experiments

All mouse experiments were approved by the University of Missouri Kansas City Institutional Animal Care and User Committee. C57 BL/6J mice (n=6, male), aged 11-12 weeks (25-35g), or 7 female of C57 BL/6J mice (20-30g) were randomly assigned to control and experimental groups. The mice were injected (intraperitoneally [I.P.] daily with resveratrol (30 mg/kg body weight) or with equal volume of DMSO (vehicle) for 10 days. 24 h following the last injection, the mice were sacrificed and tissue samples were frozen in liquid nitrogen and stored at −80°C until use. Heart/liver tissues (25-50 mg) were homogenized in 200 μl of CHAPS lysis buffer for the telomerase activity assay. For each sample, 0.04 μg heart protein (0.04 or 0.004 μg for liver protein) was used for each reaction. Tissues (25-50 mg) were homogenized in 400 μl of RIPA buffer and 20 μg proein of each sample was separated by 10% SDS PAGE for Western blot analysis.

### Statistical analysis

All data are presented as the mean±SD of at least three independent experiments. Differences between groups were assessed by the one-way analysis of variance (ANOVA). If the variances between groups were homogenous, groups were subjected to the multiple comparison Dunnett's test. *P < 0.05 was considered as significant.

## SUPPLEMENTARY MATERIAL AND FIGURES


