# Supplementary material for: A critical role of nicotinamide phosphoribosyltransferase in human telomerase reverse transcriptase induction by resveratrol in aortic smooth muscle cells
Source: Oncotarget. 2015 Mar 14;6(13):10812–24. doi: 10.18632/oncotarget.3580 (PMC4484421; doi:10.18632/oncotarget.3580)
Supplement: Supplementary file 1 [file oncotarget-06-10812-s001.pdf]

A critical role of nicotinamide phosphoribosyltransferase in human telomerase reverse transcriptase induction by resveratrol in aortic smooth muscle cells

Supplementary Material

S1. Extended Materials and Methods

RNA isolation, Semi-quantitive RT-PCR, and qPCR. Total RNA was isolated from ASM cells using the mirVana™ miRNA Isolation Kit from Life Technologies (Catalog#: AM1561) according to the supplier's instructions. RT-PCR was performed using Life Technolgies RNA PCR kit (Catalog#: 18080-044) with the following procedures: 1 µg total RNA was reverse transcribed with random primers at 37°C for 1 h followed by 95°C for 5 min and 4°C for 5 min in a 20 µl reaction volume. Each PCR reaction from the cDNA template (1 µl RT product) was performed using gene specific primers (Table 1) at 94°C for 3 min, then 35 cycles of 94°C for 30 sec, then 55°C for hTERT or 57°C for other genes for 30 sec and 72°C for 30 sec, followed by 72°C for 10 min for the final extension. Alpha-actin was used as a house-keeping gene control. PCR products were separated on a 3% agarose gel and stained with ethidium bromide (0.5 µg/ml). The gel images and pixel densities were acquired using an Alpha Imager and analyzed with the AlphaEase™ Stand Alone software (Alpha Innotech Corp., San Leandro, CA, USA).

Quantitative real-time reverse transcriptase polymerase chain reaction (qRT–PCR) was performed on a ViiATM 7 Real-Time PCR System (Life Technologies). TaqMan® Assays specific for NAMPT (H00237184_m1), hTERT (H00972656_m1), SIRT1 (H01009005_m1) and SIRT4 (H00202033) were purchased from Life Technologies and performed according to the manufacturer's instructions. An internal ACTB endogenous control (Cat #4326315E) was used in each assay. Relative fold changes in gene expression were determined by the comparative CT method (ΔΔCT) using the ViiATM 7 software.

Telomerase Activity Assay. Telomerase activity was determined using the TRAPeze telomerase detection kit (Catalog#: S7700, Millipore, Billerica, MA). The TRAP assay was performed according to the manufacturer's protocol with minor modification. Briefly, for each sample assayed, cell lysates (2 µl)

were added to a master mix (48 µl) consisting of 10×TRAP buffer (5 µ1), 50× deoxynucleotide triphosphates mix (1 µ1), TS primer (1 µl), TRAP primer mix (1 µ1), 5 units/µl Taq polymerase (0.4 µl), and distilled H20 (39.6 µl). Control cell extract containing telomerase (2 µl) was added to the positive control tube. The samples were incubated in a thermocycler at 30°C for 30 min prior to the initiation of thirty-three cycles of PCR, each cycle at 94°C for 30 s, 59°C for 30 s, and 72°C for 2 min, followed by a final extension at 72°C for 7 min. Gel loading dye (5 µ1) containing bromphenol blue (0.25% in 50% glycerol/50 mM EDTA) was added into each reaction tube. Each sample (25 µl) was separated in a 12.5% nondenaturing polyacrylamide gel in 0.5× Tris-borate EDTA buffer until the bromphenol blue migrated to 90% of the gel. The gel was stained with 1 µg/ml Ethidium Bromide (Catalog#: E-406, Amresco, Solon, OH) for 30 min and visualized under UV light.

siRNA and Recombinant Human NAMPT Transfection. siRNA specific for SIRT4 knockdown were purchased from Thermo Scientific (Pittsburgh, PA). The four siRNA-SIRT4 (ON-TARGET plus Human SIRT4 siRNA-SMART pool, Catalog#: 23409) target sequences were: UCAAAGAGUUACAGCGCGCUU, AGCGGUACUGGGCGAGAAA, CGGAUGACUUGGCGUGUCU, GGUCCCAACCUGCGUUCAA; The four siRNA-SIRT4 control target sequences were: UGGUUUACAUGUCGACUAA, UGGUUUACAUGUUGUGUGA, UGGUUUACAUGUUUUCUGA, UGGUUUACAUGUUUUCCUA; Stealth siRNA specific for NAMPT were custom ordered from Life Technologies (Grand Island, NY, USA). NAMPT (Stealth_116) siRNA sequences were: CCACCCAACACAAGCAAAGUUUAUU, AAUAAACUUUGCUUGUGUUGGGUGG. The NAMPT scrambled control sequences (Stealth_con116) were: CCACAACAACAAACGUUGAUCCAUU, AAUGGAUCAACGUUUGUUGUUGUGG.

ASM cells (3×10^5) were reverse transfected in 6 well tissue culture plates with siRNA complexed with Lipofectamine 2000 (Catalog#: 11668-019, Life Technologies) in Opti-MEM 1® (Catalog#: 31985-062, Life Technologies), according to the manufacture's recommendations. Briefly, ASM cells were transfected with either 100 nM siRNA-NAMPT or 20 nM siRNA-SIRT4. After 24 h the transfection

media was replaced with complete ASM culture medium and then incubated for another 24 h. The cells were then treated with 50 µM of resveratrol or an equal volume of DMSO as a vehicle control for 6 h.

A549 cells (3×10^5) were transiently transfected with the pCAGGS (vector), pCAGGS-NAMPT (NAMPT) and mutant NAMPT (H247E) constructs using LipofectamineTM 2000 (cat. no. 11668-019, Life Technologies) as described previously (13). 42 h later, cells were treated with 50 µM) resveratrol for another 6 h, at which time cell lysates were harvested for telomerase activity analysis.

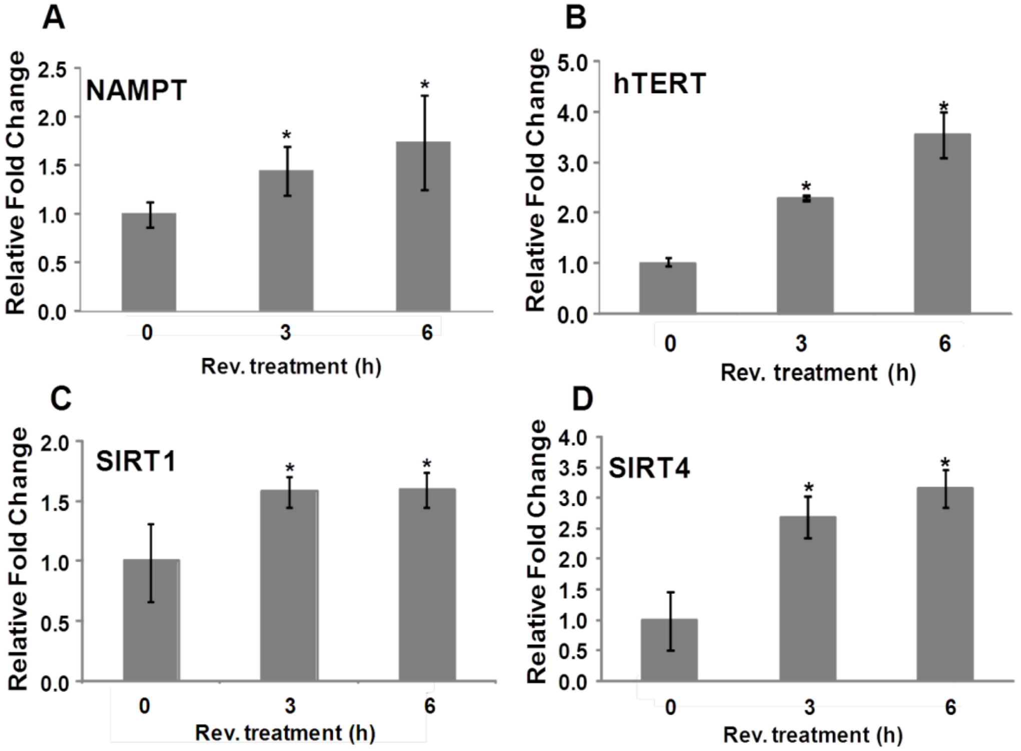

Fig. S1: Resveratrol (Rev.) induces NAMPT, hTERT, SIRT1, and SIRT4 expression in ASM cells. The mRNA expression levels of (A) NAMPT, (B) hTERT, (C) SIRT1, and (D) SIRT4 after treatment with 50 μM reseveratrol were analyzed using TaqMan® qRT–PCR gene expression assays. Relative fold changes in gene expression were determined by the comparative C_T method ($\Delta\Delta C_T$). The target genes were normalized to the ACTIN endogenous reference and then relative to the untreated controls (0 h), which served as the calibrator. Data are the relative fold change ± SD of at least three separate experiments, each analyzed by TaqMan® assays performed in quintuplicate (*$P < 0.05$).

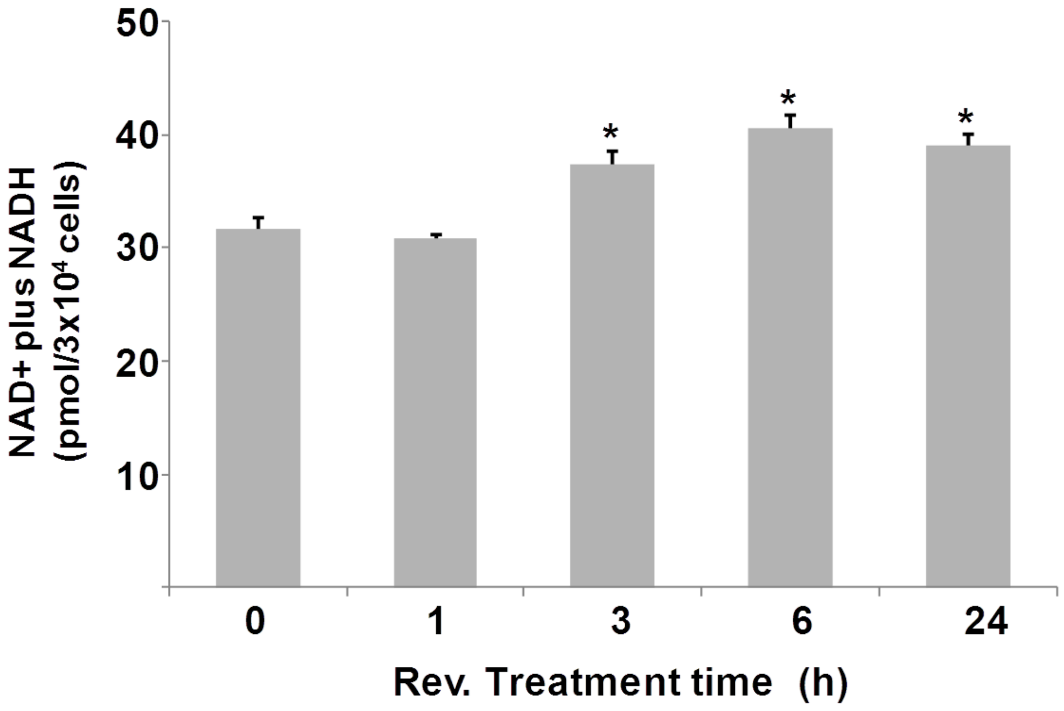

Fig. S2: Resveratrol increases NAD+ plus NADH levels in human ASM cells. ASM cells were seeded in a 96-well plate at a density of $3X10^4$ cells/well and treated with 50 μM resveratrol for indicated h. NAD+ plus NADH levels in each group of cells were assayed using NAD^+/NADH cell-based assay kit . Results are expressed as the mean+SD (n=4). * significant differences ($p<0.01$) compared with cells treated with DMSO vehicle for 6 h.

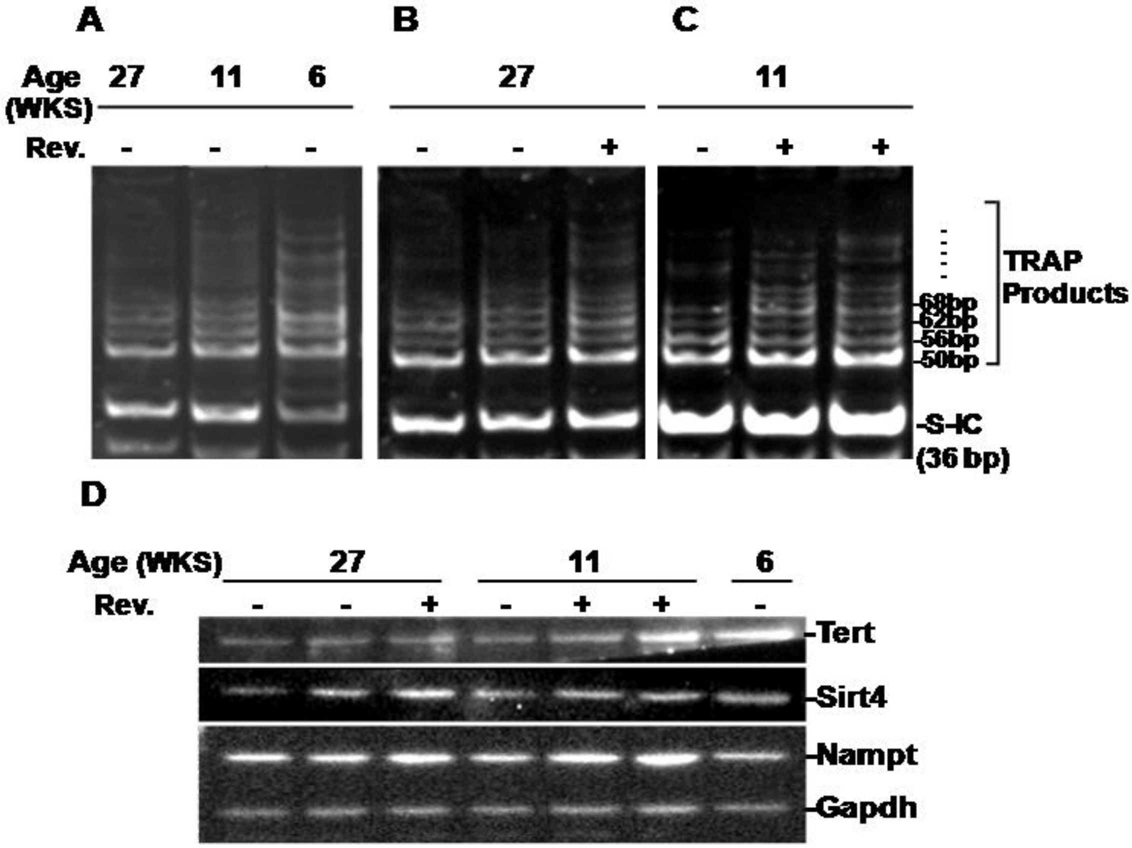

Fig. S3: Resveratrol-induced telomerase activity and TERT, Nampt, and Sirt4 in mice livers. C57 BL/6J mice at indicated ages were intraperitoneally injected with resveratrol (30 mg/kg body weight) or with DMSO (vehicle) for ten times in 11 days. Mice livers (40-100 mg) were homogenized in lysis buffer. A) Representative image of TRAP telomerase activity assay for untreated mouse liver protein. 0.04 µg protein was used in each assay. B) TRAP telomerase activity assay for age of 27 weeks group. 0.04 µg of mouse liver protein was used in each assay. C) TRAP telomerase activity assay for age of 11 weeks mouse. 0.004 µg of mouse liver protein was used in each assay reaction. D) Western blot analysis of Tert, Nampt, and Sirt4 in mice livers. 25 µg of mouse liver protein was separated by PAGE gel, transferred to PVDF membrane. Specific antibodies were used to detect Tert, Nampt, and Sirt4 expression in mice livers.

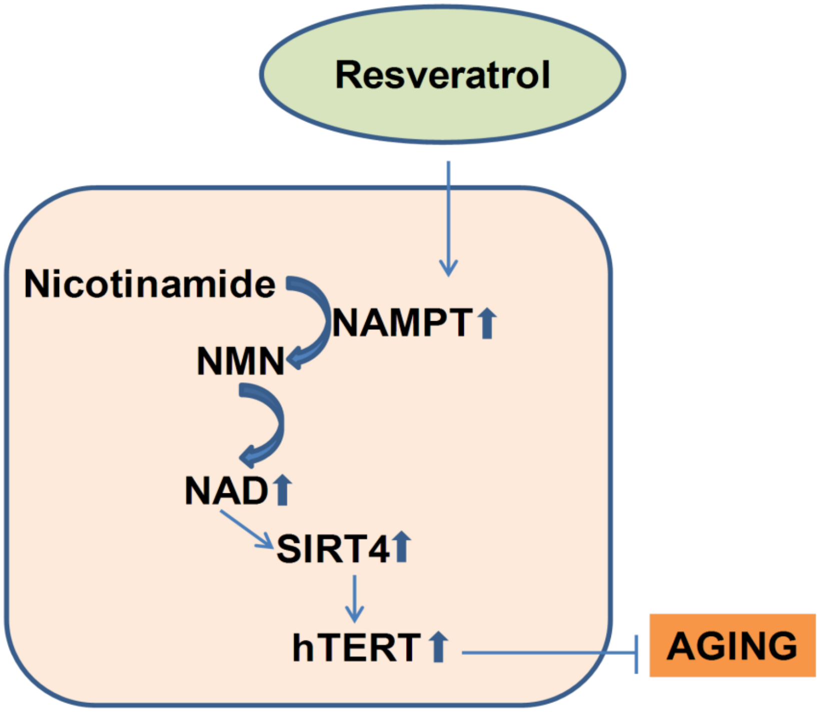

Fig. S4: Proposed signal transduction pathway of resveratrol mediated effect in human ASM cells. Our findings clearly show that resveratrol induces transcription of mRNA and a resulting increase in the protein levels of hTERT, NAMPT and SIRT4. Through the use of siRNA technology we were able to determine that resveratrol activation of hTERT is dependent upon first activating NAMPT. Activation of NAMPT results in increased recycling of NMN into NAD likely leading to increased SIRT4 activity. Activation of SIRT4 either directly or indirectly leads to an increase in hTERT that is thought to contribute to an anti-aging phenotype. This NAMPT-SIRT4-hTERT axis may represent a novel signal transduction mechanism for the anti-aging effects of resveratrol in human ASM cells.
